# Evaluation of Polyphenolic Profile and Antioxidant Activity of *Pistacia lentiscus* L. Leaves and Fruit Extract Obtained by Optimized Microwave-Assisted Extraction

**DOI:** 10.3390/foods9111556

**Published:** 2020-10-27

**Authors:** Ivona Elez Garofulić, Valentina Kruk, Ana Martić, Ivan Martić, Zoran Zorić, Sandra Pedisić, Sanja Dragović, Verica Dragović-Uzelac

**Affiliations:** 1 Faculty of Food Technology and Biotechnology, University of Zagreb, Pierottijeva 6, 10 000 Zagreb, Croatia; kruk.valentina@gmail.com (V.K.); a.oguic@gmail.com (A.M.); i.martic17@gmail.com (I.M.); zoran.zoric@pbf.unizg.hr (Z.Z.); sandra.pedisic@pbf.unizg.hr (S.P.); vdragov@pbf.hr (V.D.-U.); 2 IREKS AROMA Ltd., Trešnjevka 24, HR-10450 Jastrebarsko, Croatia; sanja.dragovic@ireks-aroma.hr

**Keywords:** *Pistacia lentiscus* L., polyphenols, antioxidant capacity, microwave-assisted extraction, UPLC/ESI-MS^2^, ORAC, phytochemicals, plant extracts

## Abstract

*Pistacia lentiscus* L. is a Mediterranean shrub known for its health promoting effects attributed to a large extent to polyphenols accumulated in all parts of the plant. Microwave-assisted extraction is a green extraction technique enabling fast and effective isolation of plant polyphenols. Therefore, the aim of this research was to optimize the microwave-assisted extraction of polyphenols from *Pistacia lentiscus* L. leaves and fruit in terms of temperature, extraction time and microwave power and to evaluate their polyphenolic profile by UPLC/ESI-MS^2^ and antioxidant capacity by ORAC assay. Optimal extraction conditions for leaf polyphenols were 69 °C, 512 W and 12 min, while for fruit were slightly more intensive—75 °C, 602 W and 15 min. Obtained total phenolic content in leaves and fruit was similar to that obtained after 30 min of the heat-reflux method. The polyphenolic profile of extracts included 34 compounds, with myricetin glycosides being the most abundant compounds among flavonoids in *Pistacia lentiscus* L. leaves and fruit and gallic acid and its derivates among the phenolic acids. ORAC assay showed higher antioxidant capacity for *Pistacia lentiscus* L. leaves extract than for fruit, which is in correlation with their respective phenolic content.

## 1. Introduction

*Pistacia lentiscus* L. is an evergreen shrub belonging to the family Anacardiaceae, widespread in the Mediterranean area. Nowadays, the plant is mostly known for its use in production of aromatic natural resin, mastic gum, used for relieving digestive problems [1]. However, all parts of the plant, including leaves, fruit, root and stems have been used in folk medicine since ancient times, due to their health benefit effects, especially as diuretics and for treatment of hypertension [2]. All those positive effects can be attributed to *Pistacia lentiscus* L. composition comprising essential oils, fatty acids and polyphenols [3]. Recent studies have shown that all parts of *Pistacia lentiscus* L. present a rich source of phenolic acids and flavonoids, mainly represented by hydroxybenzoic acids and flavonols [4,5]. These compounds are to a large extent responsible for *Pistacia lentiscus* L. antioxidant activity. This is due to polyphenols’ redox properties, which enable their use as reducing agents, hydrogen donators, metal chelators and single oxygen quenchers and consequently enable the exhibition of a wide range of positive biological effects [6]. In order to utilize the benefits of *Pistacia lentiscus* L. polyphenols, it is of a great interest to establish the proper methodology for their isolation. Effective recovery of sensitive compounds from complex and diverse parts of plant matrix (leaves, fruit) is a challenging procedure due to co-extraction of various compounds [1,7]. Conventional extraction procedures such as Soxhlet extraction are time-, solvent- and energy-consuming processes that additionally bring the risk of thermal degradation of heat sensitive polyphenolic compounds [8]. Recently, the focus has been on advanced green extraction techniques, among which the microwave-assisted extraction (MAE) is widely applied for the isolation of plant polyphenols [9,10,11]. The main advantage of MAE is the significant reduction in extraction time in comparison to conventional methods, which consequently leads to a reduction in solvent consumption and enhanced effectiveness in polyphenols isolation [12]. The heating mechanism induced by microwave irradiation causes homogenous heating of the sample and effective cell disruption due to internal superheating created by dipole rotation and ionic conduction [13], enabling the same or higher extraction yield in shorter time and at lower temperatures than conventional heat-reflux methods. The efficiency of MAE is strongly dependent on its parameters, mainly temperature, irradiation time and microwave power, used as an extraction solvent and on characteristics of the plant material itself. Accordingly, there is no unique extraction procedure for isolation of plant polyphenols, and it is of great importance to conduct its optimization regarding used plant material, targeted compounds and extraction process parameters. Apart from the isolation techniques, nowadays, there is a great demand for characterization of natural compounds present in plant material and for evaluation of their antioxidant capacity. The combination of chromatographic and spectral techniques, such as LC-MS, UPLC-MS/MS and similar provided the effective tool for characterization of plant polyphenols and enabled the insight and chemical characterization even for complex structures of flavonoid glycosides, procyanidins and tannins [14]. For determination of antioxidant capacity, several assays have been proposed that can be divided into two major groups: hydrogen atom transfer (HAT) assays (ORAC, TRAP, TOSC, CL) and single electron transfer (SET) assays (FRAP, DPPH, ABTS) [15]. Among those assays, ORAC has emerged as a method of choice for the evaluation of antioxidant capacity in food and plant material as it is relevant to radical chain-breaking capacity of antioxidants and based on the peroxyl radical which is the predominant free radical found in lipid oxidation in foods and biological systems [16]. Furthermore, it can measure both lipophilic and hydrophilic antioxidants, which makes it one of the most biologically relevant assays [17].

Therefore, the aim of this research was to study the influence of temperature, extraction time and microwave power on isolation of polyphenols of *Pistacia lentiscus* L. leaves and fruit and to establish the optimal extraction conditions for each part of the plant. Furthermore, previous studies have dealt with the partial polyphenolic profile of *Pistacia lentiscus* L. leaves or fruit, including individual polyphenolic classes [1,5,18,19,20], and to our knowledge, there is no systematic report on the polyphenolic profile of different plant parts. Hence, the intent of this research was to compare the UPLC/ESI-MS^2^ polyphenolic profile of obtained leaves and fruit extracts and to evaluate their antioxidant capacity using ORAC assay.

## 2. Materials and Methods

### 2.1. Plant Material

The samples of *Pistacia lentiscus* L. leaves and fruit were collected at the island of Korčula, Croatia (coordinates 42.961182/16.721574) and botanically identified with support of Faculty of Agriculture, University of Zagreb (Croatia). Samples of leaves and fruit were taken from the same shrub, approximately 50 kg of leaves with branches and 700 g of fruit. Samples were collected in August, in the early fruiting stage. Samples were dried at 30 °C in a laboratory drying oven (FN 500, Nuve, Ankara, Turkey) till final moisture content of 5%, milled, placed in closed plastic containers and stored at 4 °C until utilized.

### 2.2. Chemicals and Reagents

Solvents used for the extraction and analysis were HPLC grade, namely ethanol, formic acid and acetonitrile, purchased from BDH Prolabo (Lutterworth, UK). Distilled water was of Milli-Q quality (Millipore Corp., Bedford, NY, USA). Anhydrous sodium carbonate (≥99.5%) and sodium phosphate (96%) were obtained from Kemika (Zagreb, Croatia), Folin-Ciocalteu reagent from Merck (Darmstadt, Germany), fluorescein sodium salt from Honeywell Riedel-de-Haën (Bucharest, Romania), Trolox (6-hydroxy-2,5,7,8-tetramethylchroman-2-carboxylic acid) from Acros Organics (Thermo Fisher Scientific, Geel, Belgium) and 2,2′-Azobis (2-amidinopropane) hydrochloride from Sigma-Aldrich (Steinheim, Germany).

Phenolic compounds of authentic standards of caffeic, gallic, ferulic, chlorogenic and *p*-coumaric acid, quercetin-3-glucoside, kaempferol-3-rutinoside and myricetin were purchased from Sigma–Aldrich (Steinheim, Germany); epicatechin, catechin, epigallocatechin gallate, epicatechin gallate, procyanidin B1, procyanidin B2, apigenin and luteolin from Extrasynthese (Genay, France); and quercetin-3-rutinoside from Acros Organics (Thermo Fisher Scientific, Geel, Belgium).

### 2.3. Microwave-Assisted Extraction

Polyphenols from *Pistacia lentiscus* L. leaves and fruit were extracted using a single mode focused microwave reactor (Milestone, Start S Microwave Labstation for Synthesis, Sorisole, Italy) operating at 2450 MHz with adjustable microwave power. General extraction parameters were kept constant: time required to achieve extraction temperature—2 min; stirring—50%; ventilation after extraction—1 min. Solvent selection for the extraction procedures was made upon previous literature reports showing 70% ethanol to be suitable for isolation of polyphenols in similar plant material [21,22]

A mass of 1 ± 0.001 g of ground sample was mixed with 40 mL 70% aqueous ethanol solution in a round bottom flask with a magnetic stirrer and placed into a microwave reactor with a cooling system. Extraction parameters (temperature, microwave power and time) were set according to the experimental design shown in Table 1. Afterwards, extracts were cooled at the room temperature, filtered through Whatman No. 40 filter paper (Whatman International Ltd., Kent, UK), transferred into 50 mL volumetric flasks, made up to volume with solvent, transferred to plastic Falcon tubes and stored at −18 °C in nitrogen gas atmosphere until analyzed. All extractions were performed in duplicate.

### 2.4. Conventional Extraction

*Pistacia lentiscus* L. leaves and fruit polyphenols were extracted from 1 ± 0.001 g of milled sample with 40 mL 70% aqueous ethanol solution in a flat bottom Erlenmeyer flask. The mixture was extracted for 30 min with reflux, filtered through filter paper and made up to 50 mL with extraction solvent. Extracts were prepared in duplicate and stored at −18 °C in nitrogen gas atmosphere until analyzed.

### 2.5. Polyphenols Analysis

#### 2.5.1. Determination of Total Phenolic Content

Total phenolic content of *Pistacia lentiscus* L. leaves and fruit extracts was determined by the spectrofotometric Folin-Ciocalteu method described by Shortle et al. (2014) [23] with some modifications. The aliquot (100 µL) of each sample extract was mixed with 200 µL Folin-Ciocalteu reagent and 2 mL distilled water. After 3 min, 1 mL 20% sodium carbonate solution was added to the mixture. Blank was prepared according to the same procedure using the extraction solvent instead of extract. The absorbance was read at 765 nm after tempering in a water bath at 50 °C for 25 min. Total phenolic content was calculated according to the gallic acid standard calibration curve (y = 0.0035x, R^2^ = 0.9995) prepared from working standard solutions in concentration range from 50 to 500 mg/L. All measurements were performed in duplicate and results were expressed as mg gallic acid equivalents (GAE) per 100 g of sample ± standard deviation according to the gallic acid calibration curve.

#### 2.5.2. UPLC/ESI-MS^2^ Analysis

UPLC/ESI-MS^2^ analysis was performed on an Agilent series 1290 RRLC instrument (Agilent, Santa Clara, CA, USA) connected to triple quadrupole mass spectrometer (6430) with ESI ion source coupled to a binary pump, with an autosampler and thermostated column compartment. Separations were performed on a Zorbax Eclipse Plus C18 column (100 × 2.1 mm; 1.8 μm particle size) from Agilent. Column temperature was set at 35 °C, and the injection volume was 2.5 μL. The solvent composition and the gradient conditions used, as well as instrument settings, were maintained according to the method described by Elez Garofulić et al. (2018) [21]. Ionization was performed by electrospraying (ESI) in the negative and positive mode (*m/z* 100 to 1000), and the data were collected in the dynamic multiple reactions monitoring (dMRM) mode with the following parameters: positive/negative capillary voltage, 4000/3500 V; drying gas temperature of 300 °C with a flow rate of 11 L/h and nebulizer pressure 40 psi. High purity nitrogen (99.999%, Messer, Croatia) was used as inducing cone and collision gas. The MassHunter software was used for data acquisition and analysis. All measurements were performed in duplicate. An external standard calibration methodology was applied. Calibration curves were obtained by injection of six known concentrations of the following standards prepared by consecutive dilutions from a stock methanol solution: gallic acid, chlorogenic acid, caffeic acid, ferulic acid, procyanidin B1, catechin, epicatechin, epicatechin gallate, epigallocatechin gallate, quercetin-3-rutinoside, quercetin-3-glucoside, kaempferol-3-rutinoside, myricetin, luteolin and apigenin. All standards were qualified and quantified in dynamic MRM mode, using the optimized specific parameters: retention time, precursor ion, product ion, fragmentor voltage, collision energy and ionization mode. Quality parameters for the analytical method including calibration curves, instrumental detection (LOD) and quantification (LOQ) limits were reported previously [21]. Identification of phenolic compounds was carried out by comparing retention times and mass spectra with those of authentic standards. For the compounds lacking reference standards, identification was based on mass spectral data and previously reported mass fragmentation patterns, while quantification was performed as follows: feruloylquinic acid according to the ferulic acid calibration curve; *p*-coumaroylquinic acids 1 and 2 according to the *p*-coumaric acid calibration curve; 3-*O*-caffeoylquinic acid according to the chlorogenic acid calibration curve; monogalloyl glucose, 5-*O*-galloylquinic, digalloylquinic and trigalloylquinic acids according to the gallic acid; myricetin rutinoside, glucuronide and rhamnoside according to the myricetin calibration curve; kaempferol rhamnosyl hexoside, pentosyl hexoside, pentoside, rhamnoside and acetylrhamnosyl hexoside according to the kaempferol-3-rutinoside; and quercetin pentoside and rhamnoside according to the quercetin-3-glucoside calibration curve. Obtained concentrations were expressed as mg per 100 g of sample, as mean value ± standard deviation (N = 4 replicates).

#### 2.5.3. Oxygen Radical Absorbance Capacity (ORAC) Assay

The antioxidant capacity of the extracts was assessed by the oxygen radical absorbance capacity (ORAC) assay according to the study of Prior et al. (2005) [15] and Bender et al. (2014) [24] with minor modifications. The ORAC procedure used an automated plate reader (BMG LABTECH, Offenburg, Germany) with 96-well plates, and data were analyzed by MARS 2.0 software. The 2,2′-Azobis radical (2-amidinopropane) dihydrochloride (AAPH), fluorescein solution, different dilutions of 6-hydroxy-2,5,7,8-tetramethylchroman-2-carboxylic acid (Trolox) and samples were prepared in 75 µM phosphate buffer (pH 7.4). Briefly, appropriately diluted samples were added in a 96-well black plate containing a fluorescein solution (70.3 nM). The plate was incubated for 30 min at 37 °C and after the first three cycles (representing the baseline signal), AAPH (240 mM) was injected into each well to initiate the peroxyl radical generation. On each plate, different dilutions of Trolox (3.12–103.99 µM) were used as reference standard. Fluorescence intensity (excitation at 485 nm and emission at 528 nm) was monitored every 90 s over a total measurement period of 120 min. The measurements were performed in duplicate, and results are expressed as µmol of Trolox equivalents (TE) per g of sample, as mean value ± standard deviation (N = 4 replicates).

### 2.6. Experimental Design and Statistical Analysis

The experimental design and statistical analysis were performed using Statsoft STATISTICA v. 13 Experimental design software (Statsoft Inc., Tulsa, OK, USA). A central composite design (CCD) comprising 16 experimental trials with one replication of the central point was chosen to evaluate the combined effect of three independent variables—temperature, microwave power and treatment time—termed as X_1_, X_2_ and X_3_, respectively (Table 1). The operating variables were considered at three levels, namely low (−1), central (0) and high (1). Experiments were performed in duplicate, in randomized order according to the trial number as arranged by the software. Repetition experiments were carried out immediately after corresponding original experiments designed by the program. The three level values for operating variables were set as follows: for temperature at 50 (−1), 60 (0) and 70 °C (1); for microwave power at 200 (−1), 350 (0) and 500 W (1); and for extraction time at 4 (−1), 8 (0) and 12 (1) min. The parameter span taken into consideration was chosen upon previous research by Dragović-Uzelac et al. (2012) [13] showing the decrease in phenolic content of dry sage leaves after exposure to microwave irradiation at power higher than 500 W for longer than 10 min. Temperature was selected according to the boiling point of the solvent in order to avoid heat degradation changes. The response variable obtained from the experimental design was total phenolic content determined by the Folin-Cicalteau method expressed in mg GAE/g of sample. The design matrix for the experiment and the regression model for each response were calculated as follows [25]:

Y = β_0_ +∑β_i_X_i_ + ∑β_ii_X_i_^2^ + ∑β_ij_X_i_X_j_, (1)

where Y is predicted response; β_0_ is the fixed response at central point; β_i_, β_ii_ and β_ij_ are the linear, quadratic and interaction coefficients. Analysis of variance (ANOVA) was carried out to determine any significant differences (*p* < 0.5) among the applied extraction conditions. The model was fitted by multiple linear regressions (MLR). The validity of the quadratic empirical model was tested using the analysis of variance and lack of fit test. The confidence level used was 95%.

A prediction and profiling tool was applied for optimization of the microwave-assisted extraction experiment. Preference for the content of total phenols was set at high (1.0). Factors were set at optimum value and were observed as follows: temperature at 20 steps, microwave power at 100 steps and extraction time at 8 steps.

## 3. Results and Discussion

### 3.1. Microwave-Assisted Extraction Optimization

Results of total phenolic content determination in *Pictacia lentiscus* L. leaves and fruit extracts obtained at different conditions of microwave assisted extraction are shown in Table 1. It can be observed that total phenolic content in leaves ranged from 67.72 ± 1.51 to 110.18 ± 2.22 mg GAE/g, while it was significantly lower in fruit, from 22.69 ± 1.41 to 41.72 ± 2.82 mg GAE/g. These results are in accordance with previous studies by Trabelsi et al. (2016) [20] and Yemmen et al. (2017) [26] reporting the highest phenolic content of 46.07 mg GAE/g in fruit and 124.1 mg GAE/g in leaves, respectively.

ANOVA results (Table 2) showed a significant influence of extraction time on total phenolic content in leaves, while phenolic content of fruit was significantly influenced by both temperature and extraction time. Phenolic content in *Pistacia lentiscus* L. leaves extract increased with prolongation of extraction time, reaching its maximum after 12 min. However, further prolongation of treatment, in duration of 15 min, caused reduction in total phenolic content. A similar extraction trend regarding the effect of extraction time was observed for the phenolic content of *Pistacia lentiscus* L. fruit. These observations are in accordance with previous reports by other authors for different plant material. Rafiee et al. (2011) [27] reported 12 min extraction time to be optimal for microwave assisted extraction of olive leaf polyphenols and also observed a decrease in phenolic content with further prolongation of extraction time. Alara et al. (2018) [28] conducted single factor experiments for the effect of extraction time on the total phenolic content of *Vernonia amygdalina* leaves and reported the increase in extracted total phenols from 2 to 10 min, followed by reduction with further prolongation of time. These observations show that generally there is an increase in phenolic content of both leaves and fruit with prolongation of extraction time until one point when further prolongation causes reduction in phenolic yield due to degradation changes. In comparison to conventional extraction techniques, the extraction equilibrium can be reached much faster due to accelerated solubility of phenolic compounds at high temperatures and exposure to microwave irradiation, therefore limiting or avoiding the risk of significant thermal degradation [29].

Temperature did not significantly influence total phenolic content of *Pistacia lentiscus* L. leaves, whilst it significantly affected the one of fruit. For *Pistacia lentiscus* L. fruit, there was an increase in total phenolic content with temperature elevation, indicating enhanced penetration of the solvent into the material and, therefore, higher solubility and extraction rate of targeted compounds. Furthermore, temperature elevation provides better mass transfer, less surface tension and viscosity of the solvent [30]. However, prolonged exposure at elevated temperatures can lead to degradation of phenols due to different degradation processes such as oxidation, decomposition and polymerization reactions [31,32]. Therefore, temperature range analyzed in this research was moderate, from 50 to 70 °C—e.g., from 43 to 77 °C with CCD axial points—chosen in order to avoid reaching boiling point of the solvent and consequently degradation changes of heat sensitive compounds.

ANOVA results also showed that microwave power did not significantly affect neither the content of total phenols in leaves nor in fruit. Similar conclusions were made by Gao et al. (2006) [33], pointing out no significant effect of microwave power from 400 to 1200 W on flavonoid extraction from *Saussurea medusa* Maxim. However, microwave power is strongly related to temperature during microwave assisted extraction. With elevation of microwave power, there is an increase in temperature, and one of the two parameters has to be kept constant during the process. In our research, temperature was kept constant and, therefore, microwave irradiation at chosen power was applied only in short increments needed for temperature maintaining.

As all parameters of the microwave assisted extraction are interconnected, their optimization regarding the analyzed material and targeted compounds is of a great interest. Therefore, all observed extraction parameters—i.e., time, temperature and microwave power—were combined in linear, quadratic and interaction coefficients in order to obtain a regression model equation describing the dependence of the total phenolic content in *Pistacia lentiscus* L. leaves and fruit of the extraction parameters (Table 2). Validity of the obtained models was tested by lack of fit test, which was insignificant for both leaves and fruit, as well as with the coefficient of determination which was higher than 0.8 for both models, implying that the model is adequate for the prediction of total phenolic concentration in *Pistacia lentiscus* L. leaves and fruit extracts. Obtained models were used for optimization purposes in order to obtain extraction conditions which will provide the highest concentration of total phenols. Predicted concentrations were confirmed experimentally and compared with those obtained by the conventional heat reflux method in a duration of 30 min (Table 3).

Optimal conditions for extraction of total phenols from *Pistacia lentiscus* L. leaves were 69 °C, 512 W and 12 min, while for fruit they were slightly higher—i.e., 75 °C, 602 W and 15 min. Differences in optimal extraction conditions for leaves and fruit can be attributed to the plant characteristics, specifically differences in morphology and structure between fruit and leaves. These conclusions were made upon the general observation that fruits require more intensive treatment than their leaf counterparts [34]. Experimentally obtained concentrations of total phenols in leaves (108.14 ± 2.12 mg GAE/g) and fruit (41.14 ± 0.76 mg GAE/g) were almost similar to those obtained after 30 min of heat reflux extraction (108.71 ± 1.87 mg GAE/g in leaves; 42.71 ± 0.93 mg GAE/g in fruit), confirming the main advantage of microwave assisted extraction, the reduction in extraction time. A similar trend was reported by Sik et al. (2020) [35], showing the reduction in extraction time from 120 min in a maceration technique to 5 min in microwave-assisted extraction of rosmarinic acid from lemon balm, thyme and sage.

### 3.2. Polyphenolic Characterization

After establishment of the optimal extraction conditions, obtained extracts of *Pistacia lentiscus* L. leaves and fruit were analyzed by UPLC/ESI-MS^2^ in order to provide insight into their polyphenolic profile. A total of 34 different phenolic compounds were identified in leaves and fruit extracts, comprising phenolic acids, flavonols, flavanols and flavones (Table 4).

Among the phenolic acids, compounds 5, 7 and 17 were identified by comparison with authentic standards as gallic, chlorogenic and *p*-coumaric acid. Compounds 4, 6, 8 and 15 were tentatively assigned as gallic acid derivates. Compound 4 showed precursor ion at *m/z* 331 with fragment ion at *m/z* 169 produced by the loss of glucose moiety (−162 amu) suggesting the structure of monogalloyl glucose. The presence of monogalloyl glucose in *Pistacia lentiscus* L. leaves was confirmed by Bozorgi et al. (2013) [36]. Compound 6 was characterized by precursor ion at *m/z* 343 and fragment ion at *m/z* 191 due to loss of galloyl moiety (−152), implicating the structure of galloylquinic acid. Compounds 8 and 15 were assigned as digalloylquinic and trigalloylquinic acids, due to precursor ions at *m/z* 495 and *m/z* 647 and fragment ions at *m/z* 343 and *m/z* 495 produced by loss of galloyl moiety [37] Remila et al. (2015) [38] reported the presence of monogalloyl, digalloyl and trigalloylquinic acid in *Pistacia lentiscus* L. fruit, while Baratto et al. (2003) [39] identified the same compounds in leaves. According to our results, the most abundant phenolic acids in *Pistacia lentiscus* L. leaves and fruit are gallic acid and its derivates, namely digalloylquinic acid in leaves (605.88 ± 4.33 mg/100 g) and gallic acid itself in fruit (57.39 ± 0.65 mg/100 g). Similar conclusions were made by Romani et al. (2002) [40] reporting the gallic acid derivates to comprise more than 70% of total phenolic compounds in *Pistacia lentiscus* L. leaves and digalloylquinic acid to be present in the highest concentration of 2680 ± 467 mg/100 g dry extract. Meheni et al. (2016) [19] reported that gallic acid is the most abundant phenolic acid in *Pistacia lentiscus* L. fruit with concentration up to 10-fold higher than the one in leaves, which is in accordance with our results.

Compared to the hydroxybenzoic acids, hydroxycinnamic acids were present in significantly lower concentrations in both leaves and fruit. Compound 3 showed the same fragmentation pattern as compound 7 with a molecular ion at *m/z* 353 and fragment ion at *m/z* 191 corresponding to the quinic acid moiety, indicating the structure of caffeoylquinic acid. As compound 7 was identified by authentic standards as chlorogenic (3-*O*-caffeoylquinic) acid, compound 3 was assigned as 5-*O*-caffeoylquinic or neochlorogenic acid due to the higher polarity and similar fragmentation path. According to the fragmentation mechanisms reported by Clifford et al. (2003) [41], compound 1 with a molecular ion at *m/z* 367 and fragment ion at *m/z* 193 was tentatively identified as feruloylquinic acid, while compounds 2 and 11 were assigned as *p*-coumaroylquinic acids (1 and 2) due to molecular ions at *m/z* 337 and fragment ions at *m/z* 173 and *m/z* 163, respectively.

Among the flavonoids, flavonol glycosides were found to be the most abundant phenolic class in both *Pistacia lentiscus* L. leaves and fruit. Compounds 18, 23 and 26 were identified as myricetin, quercetin-3-glucoside and kaempferol-3-rutinoside by comparison with authentic standards. Compounds 16,18 and 23 were tentatively assigned as myricetin derivates due to a characteristic fragment ion at *m/z* 319. Compound 16 was characterized by a precursor ion at *m/z* 627 and fragmentation loss of rhamnose (−146 amu) and glucose (−162 amu) indicating the structure of myricetin rutinoside [42]. MS^2^ spectra of compound 18 showed a precursor ion at *m/z* 495 and fragment ion at *m/z* 319 produced by loss of glucuronyl moiety (−176 amu) corresponding to the structure of myricetin glucuronide [43]. Compound 21 was characterized by a fragment ion at *m/z* 319 and loss of rhamnose (−146 amu) and was, therefore, assigned as myricetin rhamnoside [42]. Our results showed that myricetin rhamnoside is the most abundant phenolic compound in *Pistacia lentiscus* L. leaves, present in concentrations of 1782.39 ± 10.78 mg/100 g. Fruits have the highest concentration of myricetin glucuronide, namely 251.44 ± 2.17 mg/100 g. Romani et al. (2002) [40] reported that myricetin derivates comprise more than 20% of total phenolic content in *Pistacia lentiscus* L. leaves. Adversely, Kawashty et al. (2000) [44] and Bozorgi et al. (2013) [36] reported quercetin-3-glucoside as the main flavonol in *Pistacia lentiscus* L. as well as in other *Pistacia* species.

Compounds 20, 27 and 29 were tentatively identified as quercetin glycosides due to a characteristic fragment ion at *m/z* 303. They were assigned as quercetin hexoside, quercetin pentoside and quercetin rhamnoside due to losses corresponding to hexose (−162 amu), pentose (−132 amu) and rhamnose (−146 amu), respectively [43]. Among quercetin glycosides, quercetin rhamnoside was the most abundant in leaves (130.92 ± 2.23 mg/100 g), while in fruits, quercetin-3-glucoside (156.61 ± 2.77 mg/100 g). Meheni et al. (2016) [19] reported the presence of quercetin rhamnoside in both leaves and fruit.

Compounds 24, 25, 28, 30, 31 and 33 were characterized by a specific fragment ion at *m/z* 287 corresponding to kaempferol. They were tentatively identified according the specific losses as kaempferol hexoside (−162 amu for loss of hexose), kaempferol rhamnosyl hexoside (−142 amu for loss of rhamnose and −162 amu for loss of hexose), kaempferol pentosyl hexoside (−132 amu for loss of pentose and −162 amu for loss of hexose), kaempferol pentoside (−132 amu for loss of pentose), kaempferol rhamnoside (−142 amu for loss of rhamnose) and kaempferol acetyl rhamnosyl hexoside (−42, −146 and −162 amu for losses of acetyl moiety, rhamnose and hexose) [45,46]. Among kaempferol glycosides, in *Pistacia lentiscus* L. leaves, kaempferol hexoside was determined in the highest concentration (19.44 ± 0.29 mg/100 g), while in fruit, the most abundant was kaempferol rhamnoside (2.17 ± 0.04 mg/100 g). Bampouli et al. (2014) [1] also identified kaempferol hexoside and its isomers in leaves of *Pistacia lentiscus* var. Chia.

All compounds belonging to the class of flavanols (9, 10, 12, 13, 14 and 22) were identified according to their characteristic MS^2^ spectra and by comparison with authentic standards and were, therefore, assigned as procyanidin B1, procyanidin B2, epicatechin, catechin, epigallocatechin gallate and epicatechin gallate. Catechin was the most representative flavanol in *Pistacia lentiscus* L. leaves, determined in concentration 31.70 ± 0.26 mg/100 g, while in fruit it was procyanidin B1 in a concentration of 20.69 ± 0.19 mg/100 g. Zitouni et al. (2016) [47] and Meheni et al. (2016) [19] reported the presence of catechin in leaves in a concentration of 4.106 and 31.79 mg/g dm, respectively.

Belonging to the class of flavones, compounds 32 and 34 were identified as luteolin and apigenin by comparison with authentic standards. It can be observed that concentration of luteolin was significantly higher than apigenin in both leaves and fruit extract. Their presence in *Pistacia lentiscus* L. leaves was previously reported by Bampouli et al. (2014) [1], while Meheni et al. (2016) [19] found luteolin to be the second most abundant polyphenol in fruit.

### 3.3. Antioxidant Capacity

**Figure 1:**
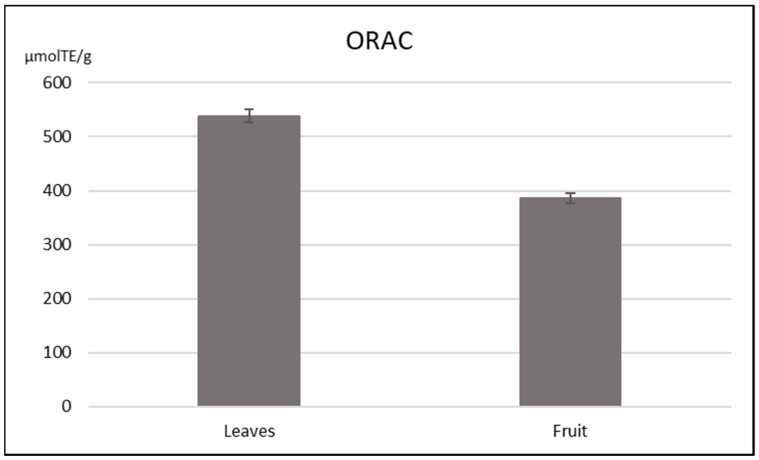
Antioxidant capacity of Pistacia lentiscus L. leaves and fruit extracts determined by ORAC assay.

In order to determine the antioxidant capacity of extracts obtained at optimized conditions, an ORAC assay was employed. *Pistacia lentiscus* L. leaves showed ORAC value of 538.41 ± 12.32 µmol TE/g, while for fruit it was lower, 386.82 ± 9.43 µmol TE/g (Figure 1).

These results are in correlation with phenolic content which was, according to our results, higher for leaves than fruit. Dahmoune et al. (2014) [3] compared the antioxidant capacity of *Pistacia lentiscus* L. leaves extract obtained by conventional extraction, ultrasound-assisted extraction and accelerated solvent extraction. They reported an ORAC value of 671.07 ± 58.80 µmol TE/g for conventional, 517.52 ± 35.18 µmol TE/g for ultrasound and 257.07 ± 20.00 µmol TE/g for accelerated solvent extraction and concluded that solvent and extraction time reduction have a negative impact on antioxidant capacity of extracts. It can be observed that ORAC values for conventional and ultrasound extraction determined in their research are in a similar range to those we obtained. Remila et al. (2015) [38] determined the ORAC value of both *Pistacia lentiscus* L. leaves and fruit extracts obtained by maceration. Similar to our findings, they also concluded that leaves show higher antioxidant capacity (442.1 µmol TE/L) than fruit (281.2 µmol TE/L). However, their ORAC values are significantly lower than the ones we reported—10,768.2 and 7736.4 µmol TE expressed per volume of extract, respectively. These differences may be attributed to the extraction method, extraction solvent and the number of phenolic compounds present in the extract, as authors applied maceration in 95% ethanol for isolation of polyphenols.

## 4. Conclusions

Microwave-assisted extraction showed to be effective method for the isolation of *Pistacia lentiscus* L. leaves and fruit polyphenols. Differences were observed between favorable extraction conditions for leaves and fruit, as optimal extraction conditions for fruit were more intensive in terms of temperature and irradiation time than for leaves. Total phenolic content in *Pistacia lentiscus* L. leaves and fruit obtained by optimized microwave-assisted extraction was similar to that obtained by conventional extraction, but achieved within a significantly shorter time, thereby confirming the MAE’s advantages in terms of reduction in the extraction time and energy consumption. Polyphenolic profile of *Pistacia lentiscus* L. comprised 34 compounds belonging to the classes of hydroxybezoic and hydroxicinnamic acids, flavonols, flavanols and flavones. Flavonols showed to be the predominant polyphenolic group in both leaves and fruit, represented by myricetin rhamnoside as the most abundant compound in leaves and by myricetin glucuronide in fruit. Among the phenolic acids, gallic acid and its derivates were present in highest concentrations, with digalloylquinic acid being characteristic for leaves and gallic acid itself for fruit. Both leaf and fruit extracts showed high antioxidant capacity presented by the ORAC value, confirming the strong potential of *Pistacia lentiscus* L. polyphenols for use as natural antioxidants and ingredients for value-added products.

## Figures and Tables

**Table 1:** Total phenolic content of *Pistacia lentiscus* L. leaves and fruit extracts obtained under different conditions of microwave-assisted extraction according to the central composite design. Results are expressed as mean values ± standard deviation (N = 4).

Sample	Temperature (°C)	Microwave Power (W)	Time (min)	Total Phenolic Content (mg GAE/g)	
				Leaves	Fruit
1	50	200	4	94.95 ± 3.43	29.54 ± 0.81
2	50	200	12	104.00 ± 4.04	33.00 ± 0.20
3	50	500	4	95.14 ± 3.43	30.57 ± 1.21
4	50	500	12	99.76 ± 0.30	37.71 ± 2.12
5	70	200	4	85.71 ± 0.2	35.86 ± 1.31
6	70	200	12	100.90 ± 1.41	41.72 ± 2.82
7	70	500	4	85.14 ± 2.63	36.78 ± 2.92
8	70	500	12	110.18 ± 2.22	38.29 ± 0.81
9	43	350	8	88.63 ± 0.91	22.69 ± 1.41
10	77	350	8	110.14 ± 3.64	34.36 ± 2.52
11	60	98	8	94.19 ± 2.42	38.14 ± 0.20
12	60	602	8	107.93 ± 2.52	34.43 ± 2.53
13	60	350	1	67.72 ± 1.51	27.00 ± 2.22
14	60	350	15	94.05 ± 5.05	40.82 ± 1.01
15 (C)	60	350	8	94.67 ± 1.21	33.50 ± 0.60
16 (C)	60	350	8	92.57 ± 3.64	34.25 ± 1.01

**Table 2:** Analysis of variance (ANOVA) for the effect of temperature, microwave power and extraction time on total phenolic content of *Pistacia lentiscus* L. leaves and fruit and polynomial fit models for their prediction.

Factor	Total Phenolic Content			
	Leaves		Fruit	
	F-Ratio	*p*-Value	F-Ratio	*p*-Value
Temperature (X_1_)	19.99	0.14	448.01	0.03 *
Microwave power (X_2_)	25.58	0.12	2.35	0.37
Time (X_3_)	320.93	0.04 *	447.32	0.03 *
X_1_X_2_	9.23	0.20	30.25	0.11
X_1_X_3_	39.99	0.10	4.64	0.28
X_2_X_3_	1.67	0.42	0.20	0.73
Lack of fit	27.29	0.14	34.70	0.13
Model	226.6418−3.9910 X_1_ + 0.0261 X_1_^2^ − 0.1614 X_2_ + 0.0010 X_2_^2^ + 0.0012 X_3_ − 0.2254 X_3_^2^ + 0.0110 X_1_X_2_ + 0.0830 X_1_X_3_ + 0.0110 X_2_X_3_		−46.7913 + 2.1914 X_1_ − 0.0131 X_1_^2^ − 0.0034 X_2_ + 0.0001 X_2_^2^ + 0.9019 X_3_ + 0.0312 X_3_^2^ − 0.0007 X_1_X_2_ − 0.0101 X_1_X_3_ − 0.0011 X_2_X_3_	
R^2^	0.83		0.87	

* *p* ≤ 0.05.

**Table 3:** Predicted and experimental concentrations of total phenolic content of *Pistacia lentiscus* L. leaves and fruit obtained at optimal conditions of microwave-assisted extraction and comparison to the 30 min conventional extraction.

Total Phenolic Content (mg/g)			Optimized Microwave Assisted Ekstraction		Conventional Extraction
			Predicted	Experimental	
Leaves	Temperature, °C	69	112.71	108.14 ± 2.12	108.71 ± 1.87
	Microwave power, W	512			
	Time, min	12			
Fruit	Temperature, °C	75	41.85	41.14 ± 0.76	42.71 ± 0.93
	Microwave power, W	602			
	Time, min	15			

**Table 4:** Mass spectrometric data and identification of polyphenolic compounds in *Pistacia lentiscus* L. leaves and fruit extracts obtained by optimized microwave-assisted extraction.

Compound	Rt, min	Cone Voltage (V)	Collision Energy (V)	Ionization Mode	Precursor Ion (*m/z*)	Fragment Ions (*m/z*)	Tentative Identification	Concentration mg/100 g	
								Leaves	Fruit
1	0.905	80	5	-	367	193	feruloylquinic acid	2.29 ± 0.09	1.38 ± 0.06
2	0.916	80	10	-	337	173	*p*-coumaroylquinic acid 1	0.21 ± 0.01	0.22 ± 0.01
3	0.918	80	10	-	353	191	5-*O*-caffeoylquinic acid	0.46 ± 0.02	0.48 ± 0.02
4	0.979	100	10	-	331	169	monogalloyl glucose	18.76 ± 0.26	15.08 ± 0.18
5	1.313	100	10	-	169	125	gallic acid *	4.34 ± 0.10	59.27 ± 0.65
6	2.144	100	10	-	343	191	5-*O*-galloylquinic acid	3.08 ± 0.12	0.60 ± 0.02
7	2.639	80	10	-	353	191	chlorogenic acid *	0.27 ± 0.01	0.22 ± 0.01
8	3.251	100	10	-	495	343	digalloylquinic acid	605.88 ± 4.33	57.39 ± 0.61
9	3.346	120	5	+	579	427	procyanidin B1 *	13.58 ± 0.19	20.69 ± 0.19
10	3.468	120	5	+	579	427	procyanidin B2 *	10.72 ± 0.15	11.93 ± 0.12
11	3.602	80	10	-	337	163	*p*-coumaroylquinic acid 2	0.33 ± 0.01	0.27 ± 0.01
12	3.935	100	10	+	291	139	epicatechin *	31.66 ± 0.31	18.28 ± 0.17
13	3.944	100	5, 10	+	291	139	catechin *	31.70 ± 0.26	18.56 ± 0.16
14	5.171	100	5, 15	+	459	139	epigallocatechin gallate *	9.16 ± 0.17	1.76 ± 0.03
15	5.208	100	10	-	647	495	trigalloylquinic acid	102.10 ± 2.09	13.59 ± 0.11
16	5.836	120	15	+	627	319	myricetin rutinoside	528.90 ± 4.27	172.63 ± 2.98
17	5.94	80	10	-	163	119	*p*-coumaric acid *	ni	0.89 ± 0.02
18	5.992	120	15	+	495	319	myricetin glucuronide	750.00 ± 6.11	251.44 ± 2.17
19	5.992	140	25	+	319	273	myricetin *	304.09 ± 2.98	14.64 ± 0.16
20	6.549	120	5	+	611	303	quercetin-3-hexoside	31.94 ± 0.23	23.94 ± 0.19
21	6.665	120	15	+	465	319	myricetin rhamnoside	1782.39 ± 10.78	24.72 ± 0.21
22	6.77	100	5, 15	+	442.9	139	epicatechin gallate *	1.47 ± 0.06	1.26 ± 0.03
23	6.886	100	5	+	465	303.1	quercetin-3-glucoside *	39.33 ± 0.40	156.61 ± 2.77
24	6.946	100	5	+	449	287	kaempferol-3-hexoside	19.44 ± 0.29	1.75 ± 0.04
25	7.309	120	15	+	595	287	kaempferol rhamnosyl hexoside	2.44 ± 0.07	1.91 ± 0.04
26	7.312	120	15	+	595	287	kaempferol-3-rutinoside *	2.41 ± 0.09	1.93 ± 0.05
27	7.342	100	5	+	435	303	quercetin pentoside	10.64 ± 0.15	1.07 ± 0.03
28	7.452	120	15	+	581	287	kaempferol pentosyl hexoside	0.21 ± 0.01	0.17 ± 0.00
29	7.702	100	5	+	449	303	quercetin rhamnoside	130.92 ± 2.23	1.90 ± 0.03
30	8.373	120	5	+	419	287	kaempferol pentoside	2.13 ± 0.09	1.60 ± 0.03
31	8.585	120	5	+	433	287	kaempferol rhamnoside	11.29 ± 0.21	2.17 ± 0.04
32	9.989	140	35	+	287	153	luteolin *	26.54 ± 0.34	1.54 ± 0.03
33	11.162	120	15	+	637	287	kaempferol acetyl rutinoside	0.20 ± 0.01	0.21 ± 0.01
34	11.185	80	30	+	271	153	apigenin *	2.02 ± 0.08	0.15 ± 0.00

* identification confirmed using authentic standards; ni/not identified.
